# Ghrelin Response to Acute and Chronic Exercise: Insights and Implications from a Systematic Review of the Literature

**DOI:** 10.1007/s40279-021-01518-6

**Published:** 2021-08-10

**Authors:** Nejmeddine Ouerghi, Moncef Feki, Nicola Luigi Bragazzi, Beat Knechtle, Lee Hill, Pantelis T. Nikolaidis, Anissa Bouassida

**Affiliations:** 1 High Institute of Sport and Physical Education of Kef, UR13JS01, University of Jendouba, 7100 Kef, Tunisia; 2 Faculty of Medicine of Tunis, Rabta Hospital, LR99ES11, University of Tunis El Manar, 1007 Tunis, Tunisia; 3 Postgraduate School of Public Health, Department of Health Sciences (DISSAL), University of Genoa, 16132 Genoa, Italy; 4 Medbase St. Gallen Am Vadianplatz, Vadianstrasse 26, 9001 St. Gallen, Switzerland; 5 Institute of Primary Care, University of Zurich, Zurich, Switzerland; 6 Division of Gastroenterology and Nutrition, Department of Pediatrics, McMaster University, Hamilton, L8S 4L8 Canada; 7 School of Health and Caring Sciences, University of West Attica, Athens, Greece

## Abstract

**Background:**

Ghrelin is a peptide hormone predominantly produced by the stomach. It exerts a wide range of functions including stimulating growth hormone release and regulating appetite, food intake, and glucose and lipid metabolism. Since physical exercise affects all these aspects, a particular interest is accorded to the relationship between ghrelin and exercise. This systematic review aimed to summarize the current available data on the topic for a better understanding of the relationship.

**Methods:**

An extensive computerized search was performed in the PubMed and SPORTDiscus databases for retrieving relevant articles. The search contained the following keywords: ghrelin, appetite-related peptides, gastrointestinal peptides, gastrointestinal hormones, exercise, acute exercise, chronic exercise, training, and physical activity. Studies investigating the effects of acute/chronic exercise on circulating forms of ghrelin were included.

**Results:**

The initial search identified 840 articles. After screening, 80 articles were included. Despite a heterogeneity of studies and a variability of the findings, the review suggests that acute exercise suppresses acyl ghrelin production regardless of the participants and the exercise characteristics. Long- and very long-term exercise training programs mostly resulted in increased total and des-acyl ghrelin production. The increase is more noticeable in overweight/obese individuals, and is most likely due to weight loss resulting from the training program.

**Conclusion:**

The review suggests that exercise may impact ghrelin production. While the precise mechanisms are unclear, the effects are likely due to blood flow redistribution and weight loss for acute and chronic exercise, respectively. These changes are expected to be metabolically beneficial. Further research is needed for a better understanding of the relationship between ghrelin and exercise.

## Key Points

**Table:** 

Ghrelin is an orexigenic gastrointestinal hormone composed of two isoforms—namely acyl and des-acyl ghrelin, which exert both analogous and opposite effects on metabolism.
Literature data on the effects of physical exercise on ghrelin production are controversial due to the heterogeneity of the studies, making an accurate interpretation of outcomes challenging.
Acute exercise is generally associated with the suppression of acyl ghrelin, while prolonged training resulted in increased total and des-acyl ghrelin, most likely due to weight loss.
Exercise-induced changes in ghrelin isoforms, namely decreased acyl ghrelin and increased des-acyl ghrelin, are expected to be metabolically beneficial.

## Introduction

Ghrelin is a peptide hormone predominantly produced by the stomach with lower amounts being synthesized in the intestine, pancreas, and in other organs [1]. Ghrelin was identified 20 years ago as an endogenous ligand able to bind to the orphan growth hormone secretagogue receptor (GHSR-1a) and to stimulate growth hormone (GH) secretion via a novel pathway [2]. Since this date, domains of actions and interest in this peptide have expanded greatly. Notably, it has been recognized to play a key role in stimulating both appetite and energy intake. Ghrelin is present in two endogenous peptide variants: acyl ghrelin (AG) and des-acyl ghrelin (DAG). The circulating AG:DAG ratio varies from 1:4 to 1:9 according to physiological factors such as body composition and nutritional status [3–5], while the underlying mechanisms are poorly understood. AG is formed by post-translational addition of a medium chain fatty acid, typically octanoate or decanoate, to the third N-terminal amino acid residue, a modification catalyzed by ghrelin-O-acyltransferase (GOAT) [6]. GOAT has been detected in the human serum, the hippocampus, and the temporal gyrus [7, 8]. AG can be locally formed by GOAT, which presumably allows it to exert effects in selected tissues and brain areas [8]. AG recognizes and binds to GHSR-1a to exert the primary hormonal and metabolic actions of ghrelin [2]. DAG derives from the proteolytic processing of unacylated proghrelin by prohormone convertase 1/3 or from cleavage of an acyl radical from AG by esterases such as butyrylcholinesterase [3, 9]. This form is unable to bind and activate the GHSR. However, it has been proven to exert diverse metabolic effects, especially on insulin sensitivity and glucose and lipid metabolism via unidentified receptors that DAG might share with AG [10, 11]. Some of these effects may modulate and even antagonize AG effects [12, 13].

Beyond the regulation of appetite, GH secretion, and the metabolism, ghrelin is involved in the modulation of gastric and gut secretion and motility, sleep–wake rhythm, and food/reward behavior [9, 14, 15]. The most well-known effect of ghrelin is the stimulation of appetite, via the activation of orexigenic hypothalamic neurocircuits, which results in an increase in body weight and adiposity. Upon fasting, hypoglycemia, or fat depletion, ghrelin is secreted into the circulation, transported across the blood–brain barrier, and binds to neurons in the hypothalamus and extra-hypothalamic regions, as well as in metabolic organs, resulting in a stimulation via diverse pathways of appetite and initiation of food intake [9]. Ghrelin’s metabolic effects have received increasing interest since pharmacological modulation of ghrelin signaling was found to be a potential therapeutic strategy to fight against diabesity and insulin resistance. Ghrelin has been proven to suppress insulin secretion [16, 17], to impair insulin sensitivity in healthy humans [18, 19], to reduce glycogen genesis and activate gluconeogenesis in the liver [20], and to stimulate glucose output from hepatocytes [21]. Mice deficient in ghrelin or in its receptor show a better glucose tolerance and insulin sensitivity [22, 23]. However, GHS-R1a antagonists enhance insulin secretion and improve glucose tolerance in diet-induced or genetically manipulated obese animals [24, 25]. Ghrelin has been proven to enhance adipogenesis, to augment fat storage enzyme activity, to increase white adipose tissue mass in selective abdominal depots (retroperitoneal and inguinal), and to reduce fat utilization/lipolysis [26–28]. In reality, the biological effects might vary according to the ghrelin isoform; the two isoforms exert either analogous or antagonist effects. Both AG and DAG induced cell survival and protected against apoptosis in animal and human pancreatic β-cells [29]. However, AG-induced suppression of insulin secretion and hyperglycemia are prevented by co-administration of DAG [18]. DAG was shown to improve insulin sensitivity [30], to promote β-cell survival and to prevent streptozotocin-induced β-cell damage [31]. AG stimulates whereas DAG inhibits glucose output from hepatocytes [21]. Both isoforms seem to stimulate lipid accumulation in human visceral adipocytes [32]. However, they exert opposing effects on medium-chain fatty acid uptake; DAG increases the uptake whereas neither AG alone nor in combination with DAG does so [33].

Ghrelin production is regulated by hormones, neurotransmitters, lipid mediators, and diverse metabolites [9]. Its secretion is entrained to habitual eating patterns in humans. Circulating levels peak before feeding, drop thereafter in parallel with ingested energy, and then decline post-prandially [34]. Not only food per se, but also its composition, influences secretion. Exposure to high glucose or long-chain fatty acids suppresses [35], whereas specific amino acids (e.g., alanine, phenylalanine, glutamate) [36] stimulate, ghrelin release. The autonomic nervous system is another regulator of ghrelin secretion. The activation of sympathetic nerves and cholinergic agonists stimulates, while adrenergic and cholinergic antagonists suppress, ghrelin secretion [37–39]. Circulating ghrelin levels are influenced by body composition and metabolism, which modulate production as well as distribution of isoforms. Circulating ghrelin concentrations negatively correlate with body mass index, being lower in obesity and higher in emaciation [40, 41]. Obesity is associated with decreased levels of DAG and increased or normal levels of AG [20, 32]. Plasma insulin and the homeostasis model assessment of insulin resistance index (HOMA-IR) correlate negatively with DAG, but positively with AG and AG:DAG ratio [20, 32].

Physical exercise is undoubtedly beneficial for health. However, the mechanisms that mediate its positive effects are not fully understood. Since both ghrelin and exercise have an impact on body composition, energy homeostasis, and glucose and lipid metabolism, interest has developed in investigating how physical exercise affects ghrelin secretion. Numerous studies investigated how ghrelin production is affected by acute and chronic exercise. The research has yielded inconsistent data, with an increase, a decrease, or no change in circulating ghrelin levels reported in response to acute or chronic exercise. Few previous reviews have summarized available data attempting to clear up the effects of exercise/training on ghrelin secretion [42–44]. They provided evidence suggesting that acute exercise transiently suppresses AG production while chronic exercise seems not to influence ghrelin levels independently of weight loss. However, an indisputable response could not be obtained due to the sparseness of data and the inconsistency of study results. On the other hand, previous reviews were not systematic and somewhat old, with the latest dating back 7 years. Since then, more studies have addressed the topic and further data on the subject have become available. In this context, we undertook a systematic literature review for an updated viewpoint on the effect of exercise/training on ghrelin production. Our purpose was to recognize how exercise affects ghrelin isoforms production and how potential changes affect the metabolic risk, and ultimately identify potential characteristics of individuals and exercise that are associated with healthy ghrelin change.

## Methods

A comprehensive systematic review was conducted and reported according to the Preferred Reporting Items for Systematic Reviews and Meta-Analysis (PRISMA) guidelines [45]. Studies eligible for inclusion were those investigating the effect of acute and chronic physical exercise on circulating ghrelin levels in humans.

### Eligibility Criteria

Studies were included if they satisfy the following criteria: (1) published in English in a peer-reviewed journal; (2) involved participants whatever their sex, age, body composition, physical fitness, and health status; (3) applied physical exercise intervention alone or combined with other intervention; (4) used acute or chronic exercise of different type, intensity, and duration; and (4) included at least two measurements (pre- and post-exercise/training) of ghrelin whatever the form detected. Studies were excluded if they: (1) did not meet the requirements of an experimental study design (e.g., reviews, case-reports, comments, opinions, editorials); (2) applied intervention with no physical exercise; (3) lacked information regarding exercise/training characteristics (e.g., type, intensity, frequency, duration); (4) used exogenous ghrelin administration; or (5) involved animals.

### Literature Search Strategy

Literature searches were conducted in PubMed and SPORTDiscus databases from inception up to December 2020. The following key terms were included and combined using the operators “AND”, “OR”: (“ghrelin” OR “appetite-related peptides” OR “gastrointestinal peptides” OR “gastrointestinal hormones”) AND (“exercise” OR “acute exercise” OR “chronic exercise” OR “training” OR “physical activity”). Further relevant studies were detected among the reference lists of the identified full-text papers, as well as through a search in similar articles and citations (PubMed database). Due to the large heterogeneity of included studies in terms of participants and exercise characteristics and the form of ghrelin detected, a systematic review and not a meta-analysis was performed.

### Study Selection

Titles identified using the literature search described above were independently screened by two authors (NO and MF) based on inclusion and exclusion criteria. Citations with potential relevance were screened at the abstract level. When abstracts indicated potential inclusion, full-text articles were reviewed. For each eligible study, relevant information was extracted including sample size, participant characteristics (i.e., sex, age class, body mass phenotype, level of fitness/training, health condition), exercise modality (i.e., acute, chronic), type (i.e., aerobic, resistance, intermittent, combined), intensity (i.e., moderate, intense) and duration, and analytical characteristics (i.e., ghrelin form detected, method of analysis, precision). The main outcome was the amount and direction of circulating ghrelin change. Changes in circulating GH and in body weight/body fat were also extracted when available. Any disagreement between the two authors in the selection of studies or the extracted data was resolved by discussion and consensus among all authors.

## Results

### Selected Studies

**Fig. 1:**
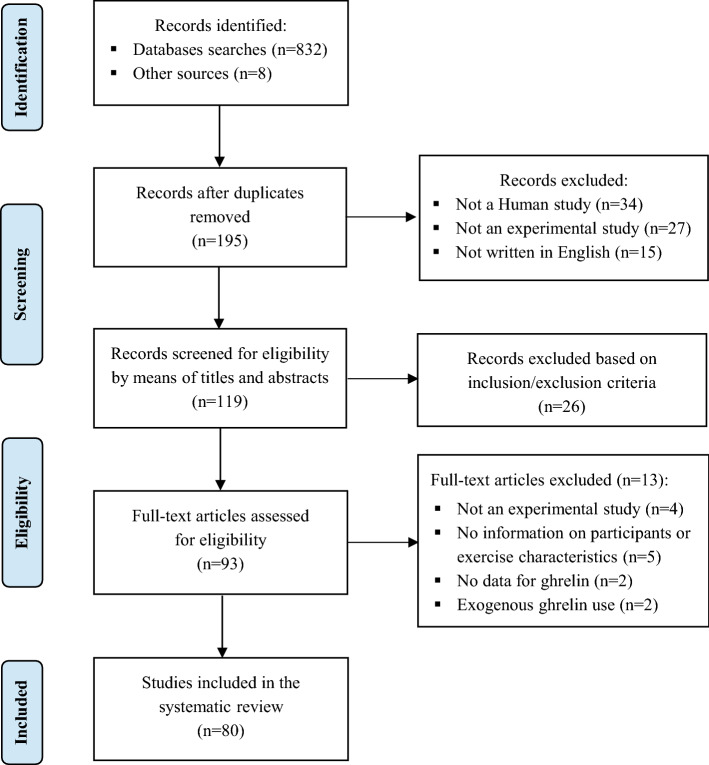
PRISMA (Preferred Reporting Items for Systematic reviews and Meta-Analysis) flow diagram of included studies

The literature search identified 840 records. After screening of titles, abstracts, and full texts, 80 relevant studies [46–125] were identified and included in the final analysis (Fig. 1). A summary of study characteristics and findings by exercise mode (acute or chronic) and duration (short-, long- or very long-term) is presented in Tables 1 and 2.

**Table 1:** Ghrelin response to short-term (< 60 min], long-term (≥ 60 min), and very long-term (≥ 90 min) acute exercise

Study, year [reference]	Participants, number, characteristics (age in years)	Exercise, type, intensity, duration	Ghrelin form, method (intra-assay CV)	Mean ghrelin change (P)	GH change
**Short-term exercise with unchanged circulating ghrelin**					
Dall et al. 2002 [46]	8 healthy adult males (40.8 ± 2.9)	Submaximal aerobic exercise, 45 min	Total ghrelin, RIA (< 5%)	DNM (NS)	Increased
	8 adult males with GH deficiency (41 ± 4.7)			DNM (NS)	Unchanged
Kraemer et al. 2004 [47]	6 well-trained male individuals, *V*O_2max_, > 52 mL·kg^−1^·min^−1^ (27.7 ± 3.20)	Run, 10 min at 60%, 10 min at 75%, 5 min at 90% and 2 min at 100% of *V*O_2max_, 27 min	Ghrelin, RIA (7.07%)	DNM (NS)	Increased
Zoladz et al. 2005 [48]	8 healthy young males (23.0 ± 0.50)	Incremental exercise (30 W every 2 min), until 60% of *V*O_2max_, 12 min	Ghrelin, RIA (5–10%)	DNM (NS)	Increased
Jürimäe et al. 2007 [49]	9 elite young male rowers (20.1 ± 3.7)	Sculling exercises, 15 min	Total ghrelin, RIA (< 10%)		
		Above the individual anaerobic threshold		+ 7.5% (NS)	Increased
		Below the individual anaerobic threshold		+ 3.8% (NS)	Increased
Erdmann et al. 2007 [50]	7 healthy young individuals (5 females, 2 males) (24.4 ± 0.6)	Cycling exercise, 100 W, 30 min	Ghrelin, RIA (10%)	− 2.3% (NS)	NM
Marzullo et al. 2008 [51]	8 obese males	Incremental exercise, 20 W every 4 min until exhaustion	Total ghrelin, RIA (4.4–10%)	− 4.1% (NS)	Increased
	8 lean males			− 21% (NS)	Increased
Unick et al. 2010 [52]	19 overweight/obese young adult females (20–37)	Walking exercise, 70–75% of HR_max_, 42 ± 8 min	Acyl ghrelin, EIA (3.9%)	DNM (NS)	NM
Thomas et al. 2012 [53]	19 young males (21.3 ± 2.3)	Resistance exercise, 6 exercises, 3 sets of 10 repetitions at 85–95% and 10 RM with 120- and + 90-s rest periods	Total ghrelin, RIA (< 5%)		NM
	9 with normal weight			DNM (NS)	
	5 with obesity class 1 (30 < BMI < 34.9)			DNM (NS)	
	5 with obesity class 2 (BMI > 35)			DNM (NS)	
Crabtree and Blannin. 2015 [54]	16 overweight individuals (6 females, 10 males) (40–60)	Treadmill walking, 60% of *V*O_2max_, 45 min (cold trial and neutral trial)	Total ghrelin, RIA (4.5%)	DNM (NS) in both cold and neutral trials	NM
			Acyl ghrelin, RIA (5.5%)	DNM (NS) in neutral trial	NM
Moraes et al. 2015 [55]	16 hemodialysis patients (11 females, 5 males) (44.4 ± 14.6)	Resistance exercise, 60% of 1 RM, 30 min	Acyl ghrelin, EIA,	− 0.9% (NS)	NM
Larsen et al. 2017 [56]	12 inactive overweight males (48 ± 5)	Strength exercise, 10 × 8 leg extensions, 75% of 1 RM, 50 min Combined aerobic (75% of *V*O_2peak_)/strength exercises, 50%/50%, 50 min	Acyl ghrelin, multiplex immunoassay	DNM (NS) DNM (NS)	NM
Ouerghi et al. 2019 [57]	7 inactive overweight middle-aged males (36.4 ± 4.35)	Moderate exercise: 60% of PAP, 20 min	Ghrelin, RIA (6%)	DNM (NS)	Increased
		Heavy exercise: 80% of PAP, 20 min		DNM (NS)	Increased
Beer et al. 2020 [58]	40 inactive healthy individuals (30 females, 10 males) (24.5 ± 7.2)	Moderate-intensity continuous exercise, 60% of *V*O_2peak_, 30 min	Active ghrelin (5.2%)	− 14.5 (NS)	NM
**Short-term exercise with decreased circulating ghrelin**					
Toshinai et al. 2007 [59]	5 inactive normal weight healthy males *V*O_2max_: 43.8 ± 0.8 ml kg^−1^ min^−1^ (26 ± 0.5)	Incremental endurance exercise at different intensities	Ghrelin, RIA		
		10 min below lactate threshold		**− **15% (*P* < 0.05)	Unchanged
		10 min at lactate threshold		**− **16% (*P* < 0.05)	Increased
		10 min at onset of lactate blood accumulation		**− **25% (*P* < 0.001)	Increased
		10 min above onset of lactate blood accumulation		**− **35% (*P* < 0.001)	Increased
Malkova et al. 2008 [60]	11 healthy, recreationally active males *V*O_2peak_, 41.8 ± 7.2 mL·kg^−1^·min^−1^ (23.8 ± 5.8)	Ergometer cycling, 90% of lactate threshold, 57 ± 3 min	Ghrelin, EIA (< 10%)	DNM (< 0.05)	NM
Balaguera et al. 2011 [61]	10 active young healthy males, *V*O_2peak_, 58 ± 7.3 ml kg^−1^ min^−1^ (19–23)	Treadmill running, 70% of *V*O_2peak_, 45 min	Acyl ghrelin	DNM (*P* < 0.05)	NM
Becker et al. 2012 [62]	82 untrained children and young adults *V*O_2max_, 55 ± 2.6 mL·kg^−1^·min^−1^ (6–30)	Cycling exercise, 70% of *V*O_2max_, 60 min	Acyl ghrelin, EIA	DNM (*P* < 0.04)	NM
Sim et al. 2014 [63]	17 inactive overweight males, *V*O_2peak_: 39.2 ± 4.8 ml kg^−1^ min^−1^ (30 ± 8)	High-intensity intermittent exercise, 15 s at 170% of *V*O_2peak_/60 s at 32% of *V*O_2peak_, 30 min,	Active ghrelin, multiplex immunoassay	DNM (*P* < 0.05)	NM
Gholipour et al. 2014 [64]	10 obese untrained young males (20.6 ± 1.4)	Intermittent treadmill exercise: 10 min, 10 min, 5 min, and 2 min at 50%, 60%, 70%, and 80% of *V*O_2max_, respectively, separated by 3 min at 3 km/h	Acyl ghrelin, EIA (6.7%)	DNM (*P* < 0.05)	Increased
Metcalfe et al. 2015 [65]	8 untrained normal weight young males, *V*O_2max_, 39 ± 10 mL·kg^−1^·min^−1^ (21 ± 2)	Continuous aerobic exercise, 50% of *V*O_2max_, 30 min	Acyl ghrelin, EIA	DNM (*P* < 0.05)	NM
		Intermittent exercise, 10 min cycling at 60 W interspersed with two “all out” sprits, 30 min		DNM (*P* < 0.05)	
Howe et al. 2016 [66]	15 endurance-trained females, *V*O_2max_, 55 ± 4.3 mL·kg^−1^·min^−1^ (18–40)	Treadmill running, 85% of *V*O_2max_, 500 kcal, 33 ± 5.6 min	Acyl ghrelin, RIA	**− **22% (*P* = 0.01)	NM
Holliday and Blanni. 2017 [67]	8 overweight individuals (4 males, 4 females) (34 ± 12)	Intermittent exercise: 4 × 30 s “flat-out” ergometer cycling interspersed with 3 min of rest	Acyl ghrelin, EIA (3.8%)	− 67% (*P* = 0.03)	NM
Larsen et al. 2017 [56]	12 inactive overweight males (48 ± 5)	Ergometer cycling, 75% of *V*O_2peak_, 50 min	Acyl ghrelin, multiplex immunoassay	DNM (*P* < 0.05)	NM
Hunschede et al. 2018 [68]	15 normal weight young males (16.1 ± 0.50)	High-intensity exercise, 75% of *V*O_2peak,_ 30 min	Active ghrelin, EIA (< 4%)	DNM (< 0.001)	NM
Matos et al. 2020 [69]	10 untrained obese males (27.6 ± 1.8)	High-intensity interval exercise, 10 × 60 s intervals at 90% of HR_max_	Total ghrelin, EIA	− 14.1% (*P* = 0.07)	NM
		Moderate-intensity continuous exercise at 50–70% of HR_max_, 20 min		− 9.6% (*P* = 0.07)	NM
Leow et al. 2020 [70]	23 physically active young individuals (10 females, 13 males) (23.6 ± 4.6)	Moderate-intensity treadmill running, 70% of *V*O_2peak_, 30 min	Active ghrelin,	DNM (*P* < 0.05)	NM
Beer et al. 2020 [58]	40 inactive individuals (30 females, 10 males) *V*O_2peak_, 26 ± 4.9 mL·kg^−1^·min^−1^ (24.5 ± 7.2)	Sprint interval t exercise, alternating 15 s at 170% of *V*O_2peak_ and 60 s at 32% of *V*O_2peak_, 30 min	Active ghrelin (5.2%)	− 42.8 (*P* < 0.001)	NM
**Short-term exercise with increased circulating ghrelin**					
Erdmann et al. 2007 [50]	7 healthy individuals (5 females and 2 males) (24.4 ± 0.6)	Cycling exercise, 50 W, 30 min	Total ghrelin, RIA (4%)	+ 8.8% (*P* < 0.05)	NM
Jürimäe et al. 2007 [71]	8 elite young male rowers	Maximal rowing ergometer test, 81% of *V*O_2max_, 20 min	Total ghrelin, RIA (< 10%)	+ 24.4% (*P* < 0.05)	Unchanged
Crabtree and Blannin. 2015 [54]	16 overweight individuals (6 females, 10 males) (40–60)	Treadmill walking, 60% of *V*O_2max_, 45 min (cold trial)	Acyl ghrelin, RIA (5.55%)	DNM (*P* < 0.05) in cold trial	NM
**Long-term exercise with unchanged circulating ghrelin**					
Burns et al. 2007 [72]	18 healthy trained subjects (9 females, 9 males), *V*O_2peak_ 57 ± 2 mL·kg^−1^·min^−1^ (24.8 ± 0.9)	Treadmill run,73.5% of *V*O_2max_, 60 min	Total ghrelin, EIA	Females, + 1.8% (NS)	NM
				Males, + 2.4% (NS)	
Martins et al. 2007 [73]	12 healthy, normal-weight individuals (6 females, 6 males) (25.9 ± 4.6)	Ergometer intermittent cycling, 65% of HR_max_, 60 min	Total ghrelin, RIA (< 10%)	DNM (NS)	NM
Sartorio et al. 2008 [74]	18 elite female athletes (25 ± 6.70)	Exercise session, 80% of *V*O_2max_, 60–90 min	Total ghrelin, RIA (6%)	− 18.9% (NS)	Increased
Hagobian et al. (2009] [75]	9 overweight young and young adult males, *V*O_2peak_, 44.9 ± 4.8 (26.8 ± 11.8)	Treadmill running, 50–65% of *V*O_2peak_ until 30% of total daily energy expenditure, 83 ± 8 min	Acyl ghrelin, RIA	DNM (NS)	NM
King et al. 2010 [76]	14 healthy young males, *V*O_2max_, 56 ± 1.8 mL·kg^−1^·min^−1^ (21.9 ± 0.5)	Submaximal treadmill walking, 60 min	Acyl ghrelin, EIA (7.8%)	DNM (NS)	NM
King et al. 2011 [77]	12 healthy physically active males (23.4 ± 1.0)	Treadmill running, 70% of *V*O_2max_, 90 min	Acyl ghrelin, EIA (7.8%)	DNM (NS)	NM
Shiiya et al. 2011 [78]	9 untrained healthy males, *V*O_2max_, 44.8 ± 1.4 mL·kg^−1^·min^−1^ (25.2 ± 0.5)	Cycling exercise, 50% of *V*O_2max_, 60 min	Total ghrelin, EIA (6.5%)	DNM (NS)	Increased
			Des-acyl ghrelin, EIA (9.8%)	DNM (NS)	Increased
Plinta et al. 2012 [79]	50 professional female basketball or handball players (21 ± 2.4)	Moderate aerobic training, 120 min (pulse 140–160/min)	Total ghrelin, EIA (6.0%)	− 1.4 (NS)	NM
		Intensive aerobic training, 90 min (pulse > 170/min)		+ 13.1 (NS)	NM
Heden et al. 2013 [80]	14 obese mildly active females, V̇O_2_peak, 49 ± 7.3 mL·kg^−1^·min^−1^ (25.1 ± 5)	Treadmill walking, 55–60% of *V*O_2peak_, 60 min	Acyl ghrelin, multiplex immunoassay (4.62%)	DNM (NS)	NM
Tiryaki-Sonmez et al. 2013 [81]	9 untrained overweight females (22.8 ± 1.38)	Treadmill exercise, 50% of *V*O_2max_, 60 min	Des-acyl ghrelin, EIA (6.5%)	DNM (NS)	NM
Douglas and Blannin. 2017 [82]	22 healthy lean females (37.5 ± 15.2)	Treadmill exercise, 60% of *V*O_2peak_, 60 min	Acyl ghrelin, EIA (5.2%)	DNM (NS)	NM
	25 overweight/obese females (45 ± 12.4)		Des-acyl ghrelin, EIA (4.8%)	DNM (NS)	
Laursen et al. 2017 [83]	11 recreationally trained males, *V*O_2peak_, 55 ± 12 mL·kg^−1^·min^−1^ (25 ± 4)	Cycling exercise, 60% W maximum, 60 min	Total ghrelin, EIA	DNM (NS)	NM
**Long-term exercise with decreased circulating ghrelin**					
Broom et al. 2007 [84]	9 trained males, *V*O_2max_, 63.3 ± 2 mL·kg^−1^·min^−1^ (21.2 ± 0.7)	Running exercise, 72% of *V*O_2max_, 60 min	Acyl ghrelin, EIA (6.6%)	DNM (*P* < 0.05)	NM
Broom et al. 2009 [85]	11 healthy male students (21.1 ± 0.3)	Aerobic exercise, 70% of *V*O_2max_, 60 min	Acyl ghrelin, EIA (4.8%)	DNM (*P* < 0.05)	NM
Shiiya et al. 2011 [78]	9 untrained healthy males, *V*O_2max_, 44.8 ± 1.4 mL·kg^−1^·min^−1^ (25.2 ± 0.5)	Cycling exercise, 50% of *V*O_2max_, 60 min	Acyl ghrelin, EIA (6.5%)	DNM (*P* < 0.05)	Increased
King et al. 2011 [86]	14 healthy normal weight males (22.0 ± 0.5)	Intermittent swimming (6 × 7 min swimming at moderate intensity interspersed with 3 min of rest), 60 min	Acyl ghrelin, EIA (6.4%)	DNM (*P* < 0.001	NM
Heden et al. 2013 [80]	14 normal-weight low-active females, *V*O_2peak,_ 50 ± 10 mL·kg^−1^·min^−1^ (26 ± 6)	Treadmill walking, 55–60% of *V*O_2peak_, 60 min	Acyl ghrelin, multiplex immunoassay (4.62%)	− 18% (*P* < 0.03)	NM
Tiryaki-Sonmez et al. 2013 [81]	9 untrained overweight females (22.8 ± 1.38)	Treadmill exercise, 50% of *V*O_2max_, 60 min followed by 60 min of rest	Acyl ghrelin, EIA (6.5%)	DNM (*P* < 0.05)	NM
Wasse et al. 2013 [87]	12 healthy active males (22.7 ± 2.3)	Running exercise, 70% of *V*O_2max_, 60 min	Acyl ghrelin, EIA (7.2%)	DNM (*P* < 0.005)	NM
		Cycling exercise, 70% of *V*O_2max_, 60 min		DNM (*P* < 0.001)	NM
Deighton et al. 2013 [88]	12 untrained healthy males, *V*O_2max_, 46.36 ± 10 mL·kg^−1^·min^−1^ (20–26)	Ergometer cycling, 65% of *V*O_2max_, 60 min	Acyl ghrelin, EIA (5.7%)	DNM (*P* < 0.05)	NM
		Interval exercise, 6 × 30 s supramaximal sprint cycling separated by 4-min recovery periods, 30 min		DNM (*P* < 0.001)	
Kawano et al. 2013 [89]	15 normal weight healthy young males, *V*O_2max_, 47.0 ± 66.2 mL·kg^−1^·min^−1^ (22–27)	Rope skipping, 3 × 10 min, 64.8–66.9% of *V*O_2max_ with 5-min interval rest, 120 min	Acyl ghrelin, EIA (8.4%)	DNM(*P* < 0.001)	NM
		Cycling 3 × 10 min at 63.9–67.5% of *V*O_2max_ with 5 min interval rest, 120 min		DNM(*P* < 0.001)	
Dorling et al. 2019 [90]	24 males with A or T allele for the obesity linked FTO rs9939609 polymorphism (21 ± 3.55)	Running exercise, 70% of *V*O_2max_, 60 min	Acyl ghrelin, EIA (4.3%)	DNM (*P* < 0.05) for both low- and high-risk alleles	NM
**Very long-term exercise with decreased circulating ghrelin**					
Ghanbari-Niaki. 2006 [91]	14 male students with recreational weight training (20.5 ± 0.5)	Resistance training (3 circuits of 10 exercises with 8–12 repetitions at 60% of 1 RM), 180 min	Total ghrelin, RIA	DNM (*P* < 0.05)	Increased
Sartorio et al. 2008 [74]	19 elite athletes of different disciplines (25 ± 6.7)	Aerobic exercise, 80% of *V*O_2max_, 60–90 min	Total ghrelin, RIA (6%)	− 20% (*P* < 0.05)	Increased
Broom et al. 2009 [85]	11 healthy male students (21.1 ± 0.3)	Resistance exercise: 10 different weight-lifting exercises, 80% of 12 RM, 90 min	Acyl ghrelin, ELISA (4.8%)	DNM (*P* < 0.05)	NM
King et al. 2010 [92]	9 non obese healthy males (18–27)	Treadmill running, 70% of *V*O_2peak_, 90 min	Acyl ghrelin, EIA (7.8%)	DNM (*P* < 0.05)	NM
Vatansever-Ozen et al. 2011 [93]	10 elite male soccer players, *V*O_2max_, 62.7 ± 5.0 mL·kg^−1^·min^−1^ (20.1 ± 0.17)	Treadmill at 50% (105 min) + 70% (last 15 min) of *V*O_2max_	Acyl ghrelin, EIA (5%)	DNM (*P* < 0.05)	NM
**Very long-term exercise with increased circulating ghrelin**					
Christ et al. 2006 [94]	11 male endurance athletes, *V*O_2max_ ≥ 60 mL·kg^−1^·min^−1^ (31.4 ± 1.7)	Cycling exercise, 50% of maximal power, 180 min	Total ghrelin, RIA (5.3%)	DNM (*P* < 0.01) in both low-fat and high-fat diet	Increased
Hagobian et al. (2009] [75]	9 overweight young and young adult females, *V*O_2peak_, 35 ± 5.2 mL·kg^−1^·min^−1^ (23.3 ± 8)	Treadmill running, 50–65% of *V*O_2peak_ until 30% of total daily energy expenditure, 88 ± 5 min	Acyl ghrelin, RIA		NM
		In deficit condition		+ 32% (*P* = 0.04)	
		In balance condition		+ 25% (*P* = 0.05)	
Jürimäe et al. 2009 [95]	9 national level male rowers (20.1 ± 1.5)	Prolonged rowing training session, 80.2 ± 1.6% of HR turn point, 120 min	Total ghrelin, RIA (< 10%)	+ 12.2% (*P* < 0.05)	Increased
Russel et al. 2009 [96]	21 healthy athletes (10 females, 10 males), *V*O_2max_ ≥ 50 mL·kg^−1^·min^−1^ (18–44)	Running at 62 ± 5% of *V*O_2max_, 90 min, followed by a 10-km time trial on a treadmill	Total ghrelin, RIA	+ 16% (o < 0.001)	Increased
Saghebjoo et al. 2013 [97]	10 male students (22.0 ± 1.32)	Circuit resistance exercise, 80% of 1 RM, 50–55 min	Acyl ghrelin, ELISA (6.2%)	+ 81.6% (*P* < 0.05)	Unchanged

*CV* coefficient of variation, *DNM* data not mentioned, *EIA* enzyme immunoassay, *GH* growth hormone, *HR* heart rate, *HR*_*max*_ maximum heart rate, *NM* not measured, *NS* not significant, *PAP* peak aerobic power, *RIA* radioimmunoassay, *RM* repetition maximum, *TV* training volume, *VO*_*2max*_ maximal oxygen uptake, *VO*_*2peak*_ peak of oxygen consumption

**Table 2:** Ghrelin response to short-term (< 12 weeks), long-term (≥ 12 weeks), and very long-term (≥ 12 months) chronic exercise

Study, year [reference]	Participants, number, characteristics (age in years)	Exercise, type, intensity, frequency, duration	Ghrelin form, method (intra-assay CV)	Mean ghrelin change (P)	Body mass or body fat change
**Short-term training with unchanged circulating ghrelin**					
Rämson et al. 2008 [98]	8 college trained male rowers (20.2 ± 1.6)	Aerobic training, volume about 10 h/wk 1	Ghrelin, RIA (< 10%)	+ 7.9% (NS)	Unchanged
		Aerobic training, volume about 15 h/wk 2		+ 0.4% (NS)	Unchanged
		Aerobic training, volume about 10 h/wk 4		+ 12.6% (NS)	Unchanged
Hedayati et al. 2012 [99]	19 healthy female students (22.2 ± 1.74)	Circuit resistance training	Total ghrelin, EIA (7.4%)		
		9 students at 40% of 1 RM, 4 wks		+ 7.7% (NS)	Unchanged
		10 students at 80% of 1 RM, 4 wks		+ 13.6% (NS)	Unchanged
Morishima et al. 2014 [100]	20 normal weight healthy sedentary individuals (33 ± 2)	Cycling exercise, 55% of *V*O_2max_, 3 times/wk, 4 wks	Active ghrelin, EIA (4.2%)		Unchanged
		Hypoxic training (n = 9), FiO_2_ = 15%)		+ 14.7% (NS)	
		Normoxic training (n = 11), FiO_2_ = 20·9%)		− 12.1% (NS)	
**Short-term training with decreased circulating ghrelin**					
Rämson et al. 2012 [101]	12 highly trained national and international male rowers (22.2 ± 3.4)	Training volume about 10 h/wk 1	Ghrelin, RIA (< 10%)		
		Training volume about 15 h/wk 2		+ 0.7 (NS)	Decreased
		Training volume about 10 h/wk 4		− 11.3 (*P* < 0.05)	Decreased
Cho et al. 2017 [102]	40 normal weight healthy females (22–28)	Intensive military training, 6 times/wk, 8 wks	Ghrelin, RIA (< 15%)	− 10% (< 0.01)	Decreased
**Short-term training with increased circulating ghrelin**					
Tremblay et al. 2019 [103]	100 inactive overweight adults/elderly with MetS (56 females, 44 males) (50–70)	High-resistance/low-aerobic training, 3 wks	Total ghrelin, EIA (1.1%)	DNM (*P* < 0.05)	Decreased
		Low-resistance/high-endurance training, 3 wks		DNM (*P* < 0.05)	Decreased
		Low-resistance/low-endurance training, 3 wks		DNM (*P* < 0.05)	Decreased
Liao et al. 2020 [104]	19 obese children (12.7 ± 1.94)	Exercise + diet intervention (moderate exercise (50–60% of HR_max_), high-intensity interval exercise (80–90% of HR_max_), and resistance training (12–15 RM), 6 times/wk, 6 wks	Ghrelin, ElA	DNM (*P* < 0.05)	Decreased
**Long-term training with unchanged circulating ghrelin**					
Jones et al. 2009 [105]	13 overweight adolescents (7 females and 5 males) (12–18)	Aerobic training, 45 min, 60–85% of *V*O_2peak_, 3 times/wk, 32 wks	Active ghrelin, EIA (9.2%)	− 6.7% (NS)	Decreased
Martins et al. 2010 [106]	22 sedentary overweight/obese individuals (14 females, 8 males) (36.9 ± 8.3)	Treadmill walking or running, 75% of HR_max_, 5 times/wk, 12 wks	Total ghrelin, RIA (< 10%)	+ 14.2% (NS)	Decreased
Guelfi et al. 2013 [107]	12 inactive overweight and obese middle-aged males (49 ± 7.0)	Aerobic training,70–80% of HR_max_, 40–60 min, 3 times/wk, 12 wks	Active ghrelin, LIA,	+ 20% (NS)	Unchanged
		Resistance training, 75–85% of 1 RM, 40–60 min, 3 times/wk, 12 wks		− 9% (NS)	Unchanged
Gibbons et al. 2017 [108]	16 inactive overweight/obese individuals (18–55)	Aerobic training, 70% of HR_max_, 5 times/wk, 12 wks	Total ghrelin, RIA (5.9%)		
		Exercising with weight loss		DNM (NS)	Decreased
		Exercising with no weight loss		DNM (NS)	Unchanged
Bowyer et al. 2019 [109]	49 normal weight older females (60–75)	Low-dose aerobic training, 50–55% of HRR, 105 ± 9 min/wk, 16 wks	Acyl ghrelin, EIA (< 10%)	+ 25% (NS)	Unchanged
Fico et al. 2020 [110]	19 inactive obese middle-aged and older adults (18 females, 1 male)	Swimming training, 20–45 min, 40–70% of HRR, 3 times/wk, 12 wks	Ghrelin	DNM (NS)	Decreased
	20 inactive obese middle-aged and older adults (18 females, 2 males)	Cycling training, 20–45 min, 40–70% of HRR, 3 times/wk, 12 wks		DNM (NS)	Decreased
**Long-term training with decreased circulating ghrelin**					
Plinta et al. 2012 [79]	50 professional female players (21 ± 2.4)	Moderate aerobic training, 120 min (pulse 140–160/min) 5 times/wk, 12 wks	Total ghrelin, EIA (6.0%)		
	15 basketball players			− 44.6 (*P* < 0.01)	Unchanged
	35 handball players			− 31.4 (*P* < 0.01)	Unchanged
Gibbons et al. 2017 [108]	16 inactive overweight/obese individuals (18–55)	Aerobic training, 70% of HR_max_, 5 times/wk, 12 wk (exerting with weight loss)	Acyl ghrelin, RIA	DNM (*P* < 0.05)	Decreased
Yu et al. 2018 [111]	39 centrally obese MetS individuals (32 females, 7 males) (58 ± 8)	Yoga training, 60 min, 5 times/wk, 52 wks	Acyl ghrelin, EIA	− 33% (vs.− 7% in controls)	Decreased
Bowyer et al. 2019 [109]	49 non-obese elderly females (60–75)	high-dose aerobic training, 50–55% of HRR, 160 ± 14 min/wk, 16 wks	Acyl ghrelin, EIA (< 10%)	− 17.6 (*P* = 0.019)	Decreased
**Long-term and very long-term training with increased circulating ghrelin**					
Leidy et al. 2004 [112]	15 normal-weight healthy females (20.2 ± 1.4)	Aerobic training 70–80% of HR_max_, 5 times/wk, 12 wks	Total ghrelin, RIA (< 2.7%)		
		5 exercising with weight stable		− 15.1 (NS)	Unchanged
		10 exercising with weight lost		+ 71% (*P* < 0.05)	Decreased
Foster-Schubert et al. 2005 [113]	87 overweight post-menopausal females (60.7 ± 6.75)	Aerobic training, 45 min, 60–75% of HR_max_, 5 times/wk, 12 wks/48 wks	Total ghrelin, RIA (3.5%)	+ 24%/ + 32% (*P* < 0.05)	Decreased
Kelishadi et al. 2008 [114]	100 obese boys and girls (7–9)	Moderate aerobic exercise, 40 min, 5 times/wk, 24 wks	Total ghrelin, RIA (9%)	DNM (*P* < 0.05)	Decreased
Mizia-Stek et al. 2008 [115]	37 middle-aged obese premenopausal females (29–52)	Cycling, 60 min, 65% of HR_max_ + diet of 1,000 kcal/day, 12 wks	Ghrelin, EIA (< 6.0%)	+ 10.5 (*P* = 0.005)	Decreased
Konopko-Zubrzycka et al. 2009 [116]	21 obese females and males (20–60)	Moderate aerobic exercise, 45-min walk, 5 times/wk, 24 wks + intragastric balloon placement + diet	Ghrelin, RIA (17.8%)	DNM (*P* < 0.01) at 4 and 24 wks	Decreased
Martins et al. 2010 [106]	22 sedentary overweight/obese individuals (14 females, 8 males) (36.9 ± 8.3)	Treadmill walking or running, 75% of HR_max_, 5 times per wk, 12 wks	Acyl ghrelin, RIA (< 10%)	+ 39% (*P* < 0.05)	Decreased
Cederberg et al. 2011 [117]	552 young undergoing military service (19.3 ± 0.9)	Intensive military training, 24 wks	Des-acyl ghrelin, EIA (11.8%)	+ 13.5% (*P* < 0.001)	Decreased
Gueugnon et al. 2012 [118]	32 obese inactive adolescents (22 females, 10 males) (14–15)	Intermittent exercise, 45–60 min, 4-min of moderate work (50% of MAP and 1 min of intense work (85% of MAP) 5 times/wk, 28 wks	Ghrelin, RIA (7.9%)	DNM (*P* < 0.05)	Decreased
Ueda et al. 2013 [119]	20 healthy mildly to moderately active females (49.1 ± 0.8)	Aerobic training, 80 min, 65% of HR_max_, 3 times/wk, 12 wks	Acyl ghrelin, EIA (< 18%)	Increased, + 11% (*P* < 0.01)	Decreased
Markofski et al. 2014 [120]	14 healthy elderly (10 females and 4 males) (71.2 ± 5)	Aerobic (60–70% of HRR) + resistance (80–85% of 1 RM) training, 20 min, 3 times/wk, 12 wks	Total ghrelin, EIA	+ 46% (*P* < 0.05)	Unchanged
Campos et al. 2014 [121]	42 post-pubertal obese adolescents (28 females, 14 males)	Aerobic training, 60 min, 3 times/wk, 52 wks	Ghrelin, EIA	+ 16.1% (NS)	Decreased
		Aerobic + resistance training, 2 × 30 min, 52 wks		+ 18.5% (NS)	Decreased
Kim et al. 2014 [122]	18 untrained healthy young males (23.6 ± 2.8)	Resistance training, 50–80 min, 60–80% of 1 RM, 6 times/wk, 12 wks	Total ghrelin, RIA (5.2%)		
		+ high-protein diet		+ 24% (*P* = 0.001)	Decreased
		+ standard diet		+ 7.5% (NS)	Unchanged
Mason et al. 2015 [123]	234 overweight/obese post-menopausal females (57.9 ± 5)	Moderate to-vigorous intensity aerobic training, 45 min, 70–85% of HR_max_, 5 times/wk, 48 wks	Total ghrelin, RIA (11.8%)	+ 1.0% (NS)	Decreased
		The same training program + diet, 48 wks		+ 7% (*P* = 0.008)	Decreased
Moraes et al. 2015 [124]	37 hemodialysis patients (16 females, 21 males) (45 ± 12.8)	Resistance training, 60–70% of 1 RM, 24 wks	Acyl ghrelin, EIA,	+ 50% (*P* < 0.05)	Unchanged
Kang et al. 2018 [125]	13 middle-aged obese females (50.1 ± 3.8)	Aerobic + resistance exercise training, 50 min, 5 times/wk, 12 wks	Ghrelin, EIA	+ 39.6% (*P* < 0.05)	Decreased
Yu et al. 2018 [111]	39 centrally obese individuals with MetS (32 females, 7 males) (58 ± 8)	Yoga training, 60 min, 3 times/wk, 52 wks	Des-acyl ghrelin, EIA	+ 14% (vs. − 27% in controls)	Decreased
Tremblay et al. 2019 [103]	100 inactive overweight adults/elderly with MetS (56 females, 44 males) (50–70)	High-resistance/low-aerobic, 12 wks/24 wks	Total ghrelin, EIA (1.1%)	DNM (*P* < 0.001)	Decreased
		Low-resistance/ high-endurance, 12 wks/24 wks		DNM (*P* < 0.001)	Decreased
		Low-resistance/low-endurance, 12 wks/24 wks		DNM (*P* < 0.001)	Decreased

*CV* coefficient of variation, *DNM* data not mentioned, *EIA* enzyme immunoassay, *FiO*_*2*_ fractional inspired oxygen, *HR*_*max*_ maximum heart rate, *HRR* heart rate reserve, *LIA* luminescence immunoassay, *MAP* of maximal aerobic power, *MetS* metabolic syndrome, *NM* not measured, *NS* not significant, *RIA* radioimmunoassay, *RM* repetition maximum, *TV* training volume, *VO*_*2max*_ maximal oxygen uptake, *VO*_*2peak*_ peak of oxygen consumption

### Participant Characteristics

Several studies included more than one arm, in which participants differed by sex, age class, body composition, training level, or applied exercise. Most studies (*n* = 57) were conducted in youth/young adults while 13, seven, and three studies were conducted in adults, elderly subjects, and children, respectively. Most studies (*n* = 41) involved males while 16 studies involved females only, and 23 studies involved individuals of both sexes. Participants were normal weight people in 46 studies, overweight/obese people in 28 studies, and both groups in two studies. Five studies involved clinical populations, including GH-deficient [46], hemodialysis [55, 124], and metabolic syndrome [103, 111] patients. Regarding fitness/training level, 33 studies involved inactive untrained subjects, 23 studies involved mildly to moderately active subjects, and 24 studies involved well-trained athletes. The sample size was particularly variable from five to 524 participants, with 77.2% of studies involving less than 30 participants.

### Exercise Characteristics

Among the 80 included studies, 51 studies investigated the ghrelin response to acute exercise, 28 studies examined the response to chronic exercise, and one study investigated the response to both acute and chronic exercise [79]. There was a high heterogeneity of studies in term of exercise/training type, intensity, frequency, and duration. Several studies used more than one type, intensity, or duration of exercise. Acute exercise studies used either aerobic (*n* = 37), resistance (*n* = 6), intermittent (*n* = 7), or combined (*n* = 6) exercise. Regarding duration, 26, 14 and 11 studies used short-term (< 60 min), long-term (≥ 60 min), and very-long term (≥ 90 min) exercise, respectively. Chronic exercise studies used either aerobic (*n* = 17), resistance (*n* = 3), combined (*n* = 8), or intermittent (*n* = 1) training programs. Most of these studies (*n* = 20) applied a long-term program (≥ 12 weeks) whereas six and three studies used a short-term (< 12 weeks) or very long-term (> 48 weeks) training program, respectively. In most acute or chronic exercise studies, the exercise was of moderate intensity. Some studies combined physical training with other interventions such as diet intervention [104, 122, 123] or intragastric balloon placement [116].

### Analytical Characteristics

Almost all studies measured one form of ghrelin while three studies measured more than one form [54, 78, 82]. Thirty-nine studies measured “total ghrelin (TG)/ghrelin”, 37 studies measured “acyl/active ghrelin”, and five studies measured DAG. In all studies, the measure was performed using high-quality immunoassay methods, generally with accurate precision.

### Response of Ghrelin According to Exercise Mode

#### Response to Acute Exercise

Of the 25 studies that examined TG/ghrelin, most studies (*n* = 15) showed no change in circulating levels. These studies involved trained [47–50, 72–74, 79, 83] or untrained [46, 74], normal weight or overweight/obese [51, 53, 57] individuals, and one study involved both healthy subjects and GH-deficient patients [46]. A lack of ghrelin change was noted with short-term, long-term, or very long-term exercise of aerobic [46–51, 57, 69, 72, 74, 79, 83], resistance [53], combined [79], or intermittent [73] type. Five studies showed a decrease in trained [60, 74, 91] or untrained [59], normal weight or overweight/obese [69] individuals following short- or long-term aerobic [60, 74], resistance [91] or intermittent [59, 69] exercise. Finally, five studies involving active subjects showed an increase in TG following short-term [50, 71] or very long-term [94–96] moderate-intensity aerobic exercise.

Thirty-six studies explored the effect of acute exercise by measuring AG. Most of these studies (*n* = 24) showed a significant decrease in circulating levels in participants with exercise of different types, intensities and durations [56, 58, 61–68, 70, 78, 80, 81, 84–90, 92, 93]. AG showed no significant change in nine studies involving either normal weight active individuals [58, 76, 77], inactive obese individuals [52, 56, 80], both lean and obese subjects [75, 82] or hemodialysis patients [55] following aerobic [52, 58, 75–77, 80, 82], resistance [55], or combined [56] exercise. Finally, three studies in either healthy active or inactive obese individuals showed an increase in AG after moderate-intensity aerobic [54, 75] or high-intensity resistance [97] exercise.

Response of DAG to acute exercise was investigated in only three studies. All these studies showed no significant change in circulating levels following moderate-intensity aerobic exercise in healthy normal weight [78] and inactive overweight/obese [81, 82] young adults. Among 16 acute exercise studies that included a GH measurement, 13 studies showed increased GH levels. However, three studies showed no variation in GH levels following acute exercise [46, 71, 97].

#### Response to Chronic Exercise

Of 21 chronic exercise studies that measured TG/ghrelin, two-thirds of studies showed an increase in circulating levels. The latter studies included children [104, 114], youth/young adults [112, 118, 122], adults [115, 116, 121, 125], or seniors [103, 113, 120, 123], who were either active normal weight [112, 120, 122] or inactive overweight/obese [103, 104, 113–116, 118, 121, 123, 125] individuals. The training was based on moderate- or high-intensity aerobic [112–116, 123], resistance [122], combined [103, 104, 120, 121, 125] or intermittent [118] exercise. An increase in ghrelin was mostly observed among physically inactive overweight/obese rather than in normal weight individuals and with long-term than short-term training programs. Five studies showed no change in circulating TG in moderately [99] or highly trained [98] subjects or inactive obese subjects [106, 108, 110] with aerobic [98, 106, 108, 110] or resistance [99] training programs of various durations. Finally, three studies involving well-trained young adults showed a decrease in TG levels following either short-term [101] or long-term [79] aerobic or short-term combined [102] exercise training.

AG was measured in ten studies using chronic exercise. Among these, three studies involving inactive or mildly active adults showed an increase in circulating levels following long-term moderate-intensity aerobic training [106, 119, 124]. Four studies showed no change in AG in either normal weight [100, 109] or overweight/obese [105, 107] subjects who underwent short-term [100] or long-term [105, 107, 109] moderate-intensity aerobic training programs. Three studies involving active normal weight [109] or obese [108, 111] individuals showed a decrease in AG with long-term or very long-term [108, 109, 111] moderate-intensity aerobic training.

Only two chronic exercise studies measured the deacylated form [111, 117]. DAG was significantly increased in active young adults undergoing a long-term combined training program [117], and in overweight/obese adults/elderly participating in a very long-term moderate-intensity aerobic training (yoga exercise) [111]. In almost all chronic exercise studies (*n* = 27), the training program resulted in a significant decrease in body mass/fat. However, some studies showed no significant body composition change with the training program [79, 98–100, 107, 109, 120, 124].

## Discussion

Despite numerous experimental and clinical data, the response of ghrelin to exercise has remained inconclusive. In this review, we summarized the currently available data aiming at advancing our knowledge on the topic. The search identified a high number of relevant studies (*n* = 80), which reported changes in circulating ghrelin levels with physical exercise as an outcome measure. The current review revealed large differences between studies in terms of participants’ characteristics, exercise protocols, and measured ghrelin forms. This high variability led to a challenging interpretation of the outcomes. Data were inconsistent, with reports showing increased, decreased, or unchanged circulating ghrelin levels with acute or chronic exercise. Since previous research revealed different responses with ghrelin to acute and chronic exercise and distinct physiological actions and possibly regulation pathways of ghrelin isoforms (i.e., AG and DAG) [3, 11–13, 42–44], the changes were examined by exercise modality (acute or chronic) and ghrelin isoform (AG, DAG or TG).

Among acute exercise studies, more than two-thirds of the studies measuring TG showed no significant change in circulating levels. However, AG levels were significantly decreased in 80% of the studies detecting this form. The suppression of AG was thought to be transient [76–78, 84, 85], but a recent powered analysis of pooled data demonstrated that acute exercise robustly suppresses circulating AG, which remains suppressed for several hours beyond the end of exercise [126]. The mechanisms underlining the exercise-induced suppression or stability of ghrelin are unclear. They reflect processes interfering with ghrelin synthesis via the modulation of GOAT or esterase activities or with its secretion into the circulation [127–129]. AG might decrease in response to augmented sympathetic output and/or gastric mucosal ischemia resulting from redistribution of blood flow from the splanchnic circulation towards the skeletal muscles during exercise [130]. It was also suggested that exercise-induced GH secretion exerts negative feedback on ghrelin production [42]. Most included studies reporting AG suppression did not include GH measurement. Only two of these studies showed an increase in GH following acute exercise [64, 78]. Further research is needed to further clarify these mechanisms.

Overall, despite discrepancies between the studies, the current review suggests that acute exercise suppresses AG while not altering TG production. However, no clear relationship has emerged between ghrelin changes, whatever the form measured, and age class, body composition, and physical aptitude of participants as well as exercise type, intensity, and duration. These findings disagree with previous studies and reviews suggesting that exercise intensity might influence AG production [44, 63, 65, 76, 77, 88]. The physiological relevance of AG suppression is uncertain. Although AG is proven to stimulate appetite and energy intake, changes in circulating AG with exercise were not strongly linked to changes in energy intake [84, 88, 92]. Other physiological or psychological factors likely have a greater effect on energy intake/food preference [44].

The review provided evidence that chronic exercise increases circulating TG and DAG production. Up to three-quarters of long-term training program studies showed an increase in circulating TG levels. A change in DAG following chronic exercise programs was rarely explored. The two studies that measured this isoform showed increased circulating levels with a long-term training program [111, 117]. The increases occurred especially in inactive overweight/obese people. Unlike TG and DAG, circulating AG levels were often unchanged or decreased. Assuming that DAG is the predominant form of ghrelin [3–5], with most studies showing no change in AG with chronic exercise, the increase in TG likely relates to the deacylated isoform. Yu et al. [117] examined the effects of a 1-year program of aerobic exercise training on both isoforms in obese subjects with metabolic syndrome. The program resulted in weight loss with an increase in DAG levels (+ 14% vs. − 27% in controls) while AG was significantly suppressed (− 33% vs. − 7% in controls).

The increase in TG/DAG associated with training programs was generally associated with weight loss [103, 104, 111, 113–118, 121, 123, 125]. So, whether the increase results from weight loss or from exercise per se is uncertain. However, TG levels were not increased when the training programs resulted in no weight loss [98–100, 107, 109]. Moreover, ghrelin levels changes are tightly correlated with changes in body mass/body fat [112, 113], which suggests that increases in TG or DAG are likely due to weight loss. In support of this hypothesis, the increases have been mostly seen in overweight/obese individuals and following long-term training programs. Weight loss is indeed more likely to occur in obese than normal weight people and in long-term rather than short-term training programs. Interestingly, Leidy et al. [112], Foster-Schubert et al. [113], and Kim et al. [122], investigating the combined effects of chronic exercise and diet, showed that TG levels only increase in participants who experienced significant weight loss. Conversely, training without significant weight loss has no impact on TG. However, other chronic exercise studies reported unchanged [108, 120, 121] or decreased [101, 102] circulating TG levels despite weight loss occurring. How changes in body mass/fat impact on circulating ghrelin is not fully understood. The changes might be due to alterations in leptin and insulin metabolism associated with weight loss. Since leptin exerts an inhibitory effect on ghrelin production [131], weight loss-induced decrease in leptin secretion results in increased ghrelin production. Accordingly, ghrelin was found to negatively correlate with leptin [132] and insulin resistance [133].

The current review failed to identify groups of individuals or types of exercise that are specifically associated with changes in circulating ghrelin. However, it suggests that prolonged high-intensity acute exercise is more effective in suppressing AG. Such exercise induces a sharper increase in sympathetic output and a more pronounced splanchnic ischemia. The review also provided evidence that long-term training is prone to increase total and des-acyl ghrelin production in overweight/obese people. This form of chronic exercise is more efficient in reducing body mass/fat, especially in obese people.

The two isoforms of ghrelin were proven to exert opposing effects on glucose and lipid metabolism and to contrarily correlate with body mass/fat [11, 13]. AG suppresses insulin secretion and stimulates glucose output from hepatocytes, while DAG exerts the opposite effects [18, 21, 22], and promotes β-cell survival and improves insulin sensitivity [30, 31]. Furthermore, obesity is associated with decreased levels of DAG and increased or normal levels of AG [20, 32]. Accordingly, high circulating AG levels are supposed to reflect increased metabolic risk, while high DAG levels and a low AG:DAG ratio would reflect low metabolic risk. Therefore, observed changes in circulating ghrelin, i.e., decrease in AG with acute exercise and increase in TG/DAG with long-term training, could be regarded as metabolically beneficial.

The current review included numerous studies covering existing literature and current knowledge in the field. This abundance of literature can be regarded as a strength since it would elicit an advance in the understanding of the impact of physical exercise on ghrelin pathways. Nevertheless, the high variability of the studies in terms of participants and exercise characteristics and ghrelin isoforms led to inconsistent results, making it difficult to draw firm conclusions. Studies involved children, youths, adults, or seniors who were either healthy normal weight or overweight/obese individuals or ill patients. Participants were physically inactive, mildly to moderately active, or highly-trained athletes. Physical intervention consisted of aerobic, resistance, intermittent, or combined exercise, of moderate- or high-intensity and variable durations. In some studies, physical exercise was combined with another intervention, especially diet. Another important source of variability was﻿ the impact of food intake on ghrelin levels. Participants exercised either in fasted or fed states with pre-exercise meals varying in caloric content, macronutrient composition, and the time at which they were provided. Diverse combinations of these factors and differences in body composition, food intake, sample collection/processing, timing of blood draw versus eating and exercising, and assay procedure may have influenced the results, making it difficult to compare outcomes of different studies.

By increasing permeability and hydrostatic pressure, acute exercise induces shifts of plasma water to the extravascular space. This causes false changes in ghrelin levels, which could confound the specific effect of exercise. Several studies in this review did not adjust for plasma volume shifts, which could have prevented an accurate estimate of ghrelin change. Since plasma shift normally causes an increase, not a decrease, in levels, AG decrease in most acute exercise studies is likely independent of these changes. Some studies did not implement a non-exercise control group, making it impossible to determine whether outcomes were solely related to exercise. Finally, small sample sizes in several studies and large between-individual variation in ghrelin levels may have underpowered some studies and prevented recognition of significant effects.

Unlike previous reviews, the current review is systematic, exhaustive, and not selective. It offers a comprehensive up-to-date view on the topic. Although the findings broadly agree with previous reviews, the review does present some novelties. It establishes that AG suppression persists beyond the end of acute exercise and is independent of exercise intensity, contrary to what has been assumed previously. It claims that increases in TG and DAG with chronic exercise are more noticeable in overweight/obese people and wih long-term programs. Novelties include the response of DAG to exercise, which was proven to increase with chronic but not acute exercise. Finally, the review findings allowed prediction of potentially beneficial ghrelin changes.

## Conclusions

The present review revealed a high variability in studies in terms of participants and exercise characteristics, as well as other factors, such as food intake, that could affect ghrelin level outcomes. This makes it challenging to interpret the data. However, the review provides evidence that acute exercise suppresses AG regardless of participants and exercise characteristics. The suppression, initially considered as transient, seems to persist beyond the end of exercise. The mechanisms responsible for the change remain hypothetical. Their relevance is unclear since the effect on appetite and energy intake seems to be absent or modest. The review suggests that chronic exercise is rather associated with increased production of TG and DAG, especially with prolonged exercise training programs and among overweight/obese individuals. These changes are likely due to weight loss rather than exercise per se. Predominant ghrelin changes (i.e., decreased AG and increased TG/DAG) are expected to reduce metabolic risk since they are supposed to reduce energy intake and fat accumulation, and enhance insulin secretion and sensitivity. Nevertheless, the results should be considered with caution given a great variability in the literature data and methodological limitations in some studies. Further research is needed for a better understanding of the effects of physical exercise on ghrelin production and metabolism. Future well-controlled trials should involve homogeneous groups of participants and use well-defined physical interventions while measuring the two isoforms of ghrelin.

## Declarations

### Data availability statement

Data sharing is not applicable to this article as no datasets were generated during the current study.
